# Preparation and Characterization of Silymarin Gel: A Novel Topical Mucoadhesive Formulation for Potential Applicability in Oral Pathologies

**DOI:** 10.3390/gels9020139

**Published:** 2023-02-07

**Authors:** Divyambika Catakapatri Venugopal, Reshma Devi Senthilnathan, Saba Maanvizhi, Yasasve Madhavan, Sathasivasubramanian Sankarapandian, Vijayalakshmi Ramshankar, Mangathayaru Kalachaveedu

**Affiliations:** 1 Department of Oral Medicine and Radiology, Sri Ramachandra Institute of Higher Education and Research (DU), Porur, Chennai 600116, India; 2 Department of Pharmacognosy, Sri Ramachandra Institute of Higher Education and Research (DU), Porur, Chennai 600116, India; 3 Department of Preventive Oncology (Research), Cancer Institute (WIA), Adyar, Chennai 600020, India; 4 Herb and Food Division, Isha Arogya, Coimbatore 641010, India

**Keywords:** silymarin, mucoadhesive gel, topical formulation, drug release, bioavailability

## Abstract

*Silybum marianum* has been used for centuries by herbalists and physicians to treat different forms of liver diseases. It contains flavonoid, which has antioxidant, anti-inflammatory, antifibrotic and anticancer properties. The objective of this research was to develop a silymarin-based mucoadhesive gel for prolonged release in oral mucosa and to evaluate the same by using *in vitro* drug release kinetic models and *ex vivo* methods for drug permeation using chicken buccal mucosa. The mucoadhesive gel was formulated in different trials by varying the concentration of silymarin and polymer. Out of 10 formulation trials, the F10 optimized trial was characterized for *in vitro* physicochemical parameters such as pH, homogeneity, viscosity, stability, drug content, *in vitro* drug release, *in vitro* antioxidant assay and *ex vivo* permeation study. Trial 10 was chosen as the best trial formulation among the other trials and was marked as an optimal trial. The physicochemical properties observed were pH to be 6.4 ± 0.01, the gel free of lumps, spreadability of 23.75 ± 0.03 and drug content of 32.77 ± 0.20 mg/g. It had no physiological changes such as color shift or fluid exudate segregation after 6 months of storage at room temperature. *In vitro* drug release established the presence of a non-fickian mechanism and demonstrated dose-dependent antioxidant activity. *Ex vivo* findings indicated 21.97 ± 0.18% release, proving that the gel can permeate through the oral mucosal membrane. Our future research will concentrate on expanding the therapeutic scope by developing the formulation trial F10 to a nanoformulation and conducting clinical trials for its potential use in various oral diseases.

## 1. Introduction

Silymarin is a flavonolignan derived from milk thistle that contains silibinin, isosilibinin, silychristin, isosilychristin, and silydianin, as well as taxifolin. Amongst its various constituents, silybin with its two diastereoisomeric compounds, namely silybin A and silybin B, are present in a higher percentage (around 70%) and contribute to the biological effect exerted by silymarin [1]. Silymarin has been used as a therapeutic agent in an array of liver disorders such as chronic liver disorders, cirrhosis, hepatocellular carcinoma, alcohol abuse, non-alcoholic fatty liver disease, virus-related liver damage and end stages of different hepatopathies because of its inherent antiviral, antioxidant, anti-inflammatory, and antifibrotic properties [2]. Silymarin has also demonstrated chemopreventive action and antimetastatic activity in *in vitro* and *in vivo* conditions in various cancers, chiefly gastrointestinal cancers and also in other cancers such as colorectal and pancreatic cancers [3]. Silymarin-mediated apoptosis, through increased cleavage of caspase 8, has been demonstrated using *in vitro* studies using oral cancer cell lines [4]. Silymarin has been found to have a good safety profile without toxicity, even after prolonged administration [5]. Although there has been a long history of silymarin use in various systemic forms such as tablets, capsules, and suspension, currently randomized controlled trials (RCT) using a topical formulation of silymarin have been employed as a therapeutic option.

Gels offer certain advantages over other formulation types, including quicker drug release, smoother delivery, and better bio-compatibility and mucoadhesivity. Gels permit adherence to the oral mucosa at the site of the lesion and rapid elimination by regular catabolic routes [6]. A topical gel formulation for skin, prepared by Sampatrao and the team showed drug content, pH, and spreadability of the formulation to be 96.6%, 6.8 and 25.45 gm.cm/s, respectively, with a maximum drug release of 96.30% throughout 3 h. The gel demonstrated pseudoplastic flow properties without showing any acute skin irritancy [7]. A randomized, double-blinded, placebo-controlled clinical trial evaluated the preventive effect of a silymarin 1% gel in comparison with a placebo, on the occurrence of radiodermatitis in breast cancer patients. The results demonstrated a significant delay in radiodermatitis development and progression when treated with silymarin [8]. Unlike skin formulations, oral gels should possess good mucoadhesion, without getting easily washed off by saliva, high water content and low surface friction for better retention in the injured oral mucosa [9]. Although currently, research is ongoing on the utility of topical skin formulation of silymarin, there is no evidence of oral mucoadhesive gel formulation for therapeutic use in various oral diseases. The reason could be attributed to poor water solubility and low bioavailability [2]. However, with the introduction of complexing with phosphatidylcholine, which has better absorption, and new silibinin glyco-conjugates (gluco, manno, galacto, and lacto-conjugates), which both have a high solubility in the water, this may be overcome [10].

Silymarin as an oral topical formulation can aid in the treatment of many oral diseases, taking advantage of its antioxidant, anti-inflammatory, antifibrotic and anticancer activity. To the best of our knowledge, since there is no oral topical formulation available in the literature, the current study aims to design, formulate and evaluate the preclinical release studies of silymarin-based mucoadhesive oral topical gel.

## 2. Results

### 2.1. Characterization of Silymarin Gel Formulation

**Figure 1:**
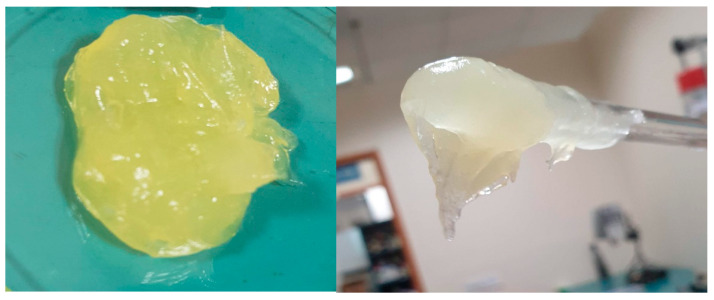
Silymarin mucoadhesive gel.

The following findings were obtained from the characterization studies, which gave an overview of the physical properties of the chosen silymarin gel formulation (F10) (Figure 1 and Table 1). The various gel formulations tested in various compositions have been listed in Table S1.

The scanning range ultraviolet spectrophotometric analysis was performed in distilled water, and 287 nm was used as the experimental value of maximum. The absorbance value of standard concentrations of 1–5 μg/mL was plotted, and linearity was observed for silymarin when analyzed at 287 nm with an R^2^ = 0.9962.

### 2.2. FTIR Spectral Analysis

**Figure 2:**
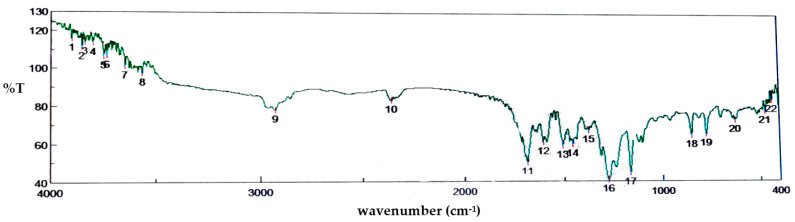
FTIR spectra of the formulation ingredients such as silymarin, carbopol and other excipients.

The FTIR spectra of the pure drug showed prominent peaks at 3566.7 cm^−1^ due to O-H stretch, 2925.48 cm^−1^ due to C-H stretch, 2.360.44 cm^−1^ due to absorption of carbon dioxide, 1716.34 cm^−1^ due to C=O stretch cyclic ketone, 1684.52 cm^−1^ due to C=C stretch aromatic, 1508.06 cm^−1^ due to C-H bending, 1281.47 cm^−1^ due to C-OH stretch, and 1166.72 cm^−1^ due to C-O stretch. From the spectra (Figure 2), it was observed that there was no significant change in the original peak of the drug and the polymer when compared with the spectra of the physical mixture of the formulated gel. This indicates that there was no interaction between drug, polymer and other excipients.

### 2.3. Evaluation Parameters

#### 2.3.1. pH

The standard pH range of the oral mucosa is 6.0 to 7.0 and the gel pH values were found to be 6.4.

#### 2.3.2. Homogeneity

There were no lumps or grittiness in any of the gel formulations that were made. (Table 2).

#### 2.3.3. Spreadability

After 1 min, the spreadability of the gels ranged from 10.73 ± 0.01 mm to 23.75 ± 0.03 mm. The gel formulations were prepared with Carbopol 934, and hence demonstrated high spreadability and extrudability (Table 2).

#### 2.3.4. Drug Content Uniformity

The percentage drug content of all formulations ranged from 0.2 ± 0.01% mg/g to 32.77 ± 0.20% mg/g (Table 2) The drug content was consistent across all gels.

#### 2.3.5. Stability

Physiological changes such as change in color, or segregation of fluid exudates of the gels were not observed after 6 months of stability studies. The pH of all the gels was unaffected and ranged from 6.5 ± 0.03 to 7.0 ± 0.06. After 6 months, and the drug content ranged from 0.17 ± 0.19 to 28.00 ± 0.004% mg/g (Table 2). The results are expressed as mean ± SD, where *n* = 3 and SD stands for standard deviation.

### 2.4. In Vitro Drug Diffusion Studies

**Figure 3:**
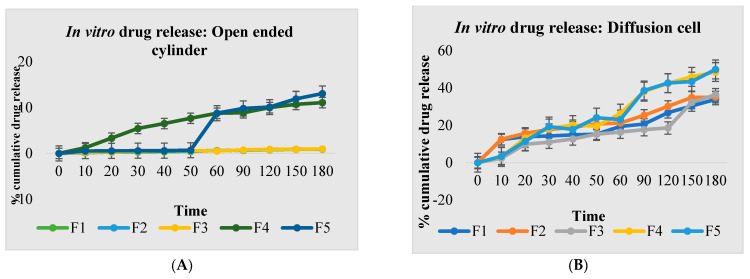

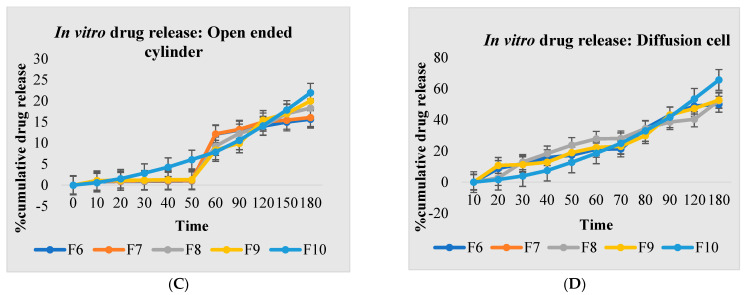
Percent cumulative drug release from gel formulations. (**A**) Open−ended cylinder (F1 to F5), (**B**) diffusion cell apparatus (F1 to F5), (**C**) Open−ended cylinder (F6 to F10), (**D**) diffusion cell apparatus (F6 to F10).

Cumulative percent drug release ranged from 21.97 ± 0.02% for the open-ended cylinder and cumulative percent drug release ranged from 65.71 ± 0.04% for the diffusion cell apparatus across all formulations (Figure 3). As a result, F10 was chosen as the optimal formulation.

### 2.5. Release Kinetics

**Figure 4:**
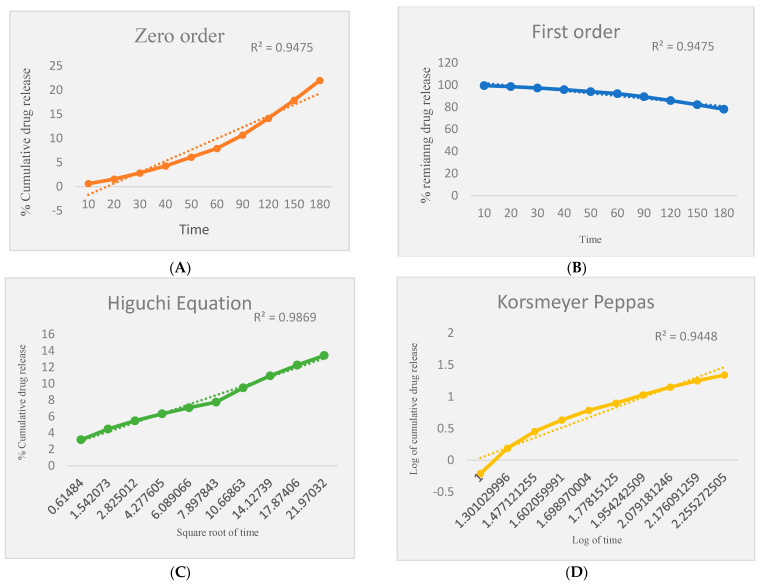
Higuchi’s kinetics drug release in the formulation. (**A**) Zero order, (**B**) first order, (**C**) Higuchi equation, (**D**) Korsmeyer Peppas.

Release kinetics of the optimized formulation F10 were investigated using zero-order, first-order, and Higuchi diffusion (Table 3 and Figure 4). Zero-order kinetics is defined as a constant amount of drug that is eliminated per unit of time, but the rate is independent of the concentration of the drug. First-order kinetics describes the constant proportion of the drug that is eliminated per unit of time. The rate of elimination is proportional to the amount of drug in the body. Higuchi’s kinetics dominated drug release in the formulation and proves that the gel is a controlled drug delivery system. It also confirms a non-Fickian mechanism. Non-Fickian drug release refers to the drug being released from the gel through a diffusion mechanism as well as another method known as chain relaxation.

### 2.6. In Vitro Antioxidant Study

**Figure 5:**
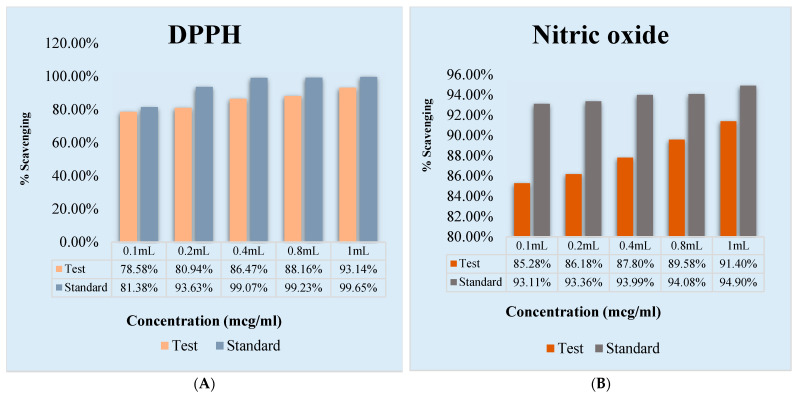
In vitro antioxidant study for optimized gel F10. (**A**) DPPH activity, which shows that it is dose-dependent, (**B**) nitric oxide, the scavenging activity.

The optimized formulation F10 was subjected to anti-oxidant analysis. The antioxidant activity of samples were investigated at various concentrations and standards (ascorbic acid). By scavenging DPPH (free radical) and converting it to DPPH, the samples demonstrated strong antioxidant activity (Figure 5). Nitric oxide (NO) is a quite unstable species that further, under aerobic conditions, reacts with O_2_ to obtain uniform nitrate and nitrite products using NO_2_, N_2_O_4_ and N_3_O_4_ intermediates. In this study, the nitrite developed by the reaction mixture was limited.

### 2.7. Ex Vivo Permeation Study

**Figure 6:**
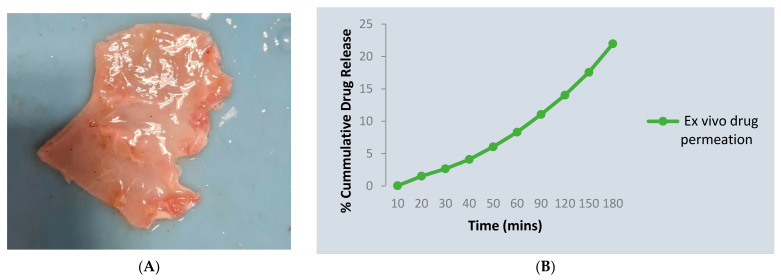
Ex vivo permeation study for the optimized formulation F10. (**A**) Chicken buccal mucosa, (**B**) Cumulative drug release.

The gel can permeate easily across the mucosal membrane. The permeation correlation coefficient range was 0.9586 and the percentage was found to be 21.97% in 3 h. The result of the study is shown in (Table 4 and Figure 6).

## 3. Discussion

Mucosal drug delivery systems have the superior advantage of intimate contact between the diseased mucosa and the drug, localizing the drug to the specific site and better patient compliance, compared to systemic drugs. Mucoadhesive forms also offer better plasma concentration and therapeutic efficacy [11]. The pre-formulation phase or study helps to lay down the foundation for transforming a new drug into a pharmaceutical formulation in such a way that it can be administered in the right manner and optimum dose. Moreover, pre-formulation studies provide better stability to the formulation by developing a proper design with adequate constituents, protecting the drug component from environmental conditions and evaluating the performance of the developed formulation to bring it to translational use in pharmacological practice [12]. Among various topical formulations such as ointments, creams, pastes, emulsions and gels, pastes and gels are the preferred topical formulations for the oral mucosa, because of their superior retentivity and mucoadhesive property. Gels, in particular, have higher water content and exhibit lower friction and hence are better retained in the injured sites of the oral cavity for a prolonged period, thereby improving the drug’s therapeutic efficacy [9].

Silymarin is a commonly used therapeutic drug with proven anti-inflammatory, antioxidant, anticancer and anti-fibrotic activities. Hence, the silymarin-based topical oral mucoadhesive gel could be a promising medication for inflammatory diseases of oral mucosa such as oral ulcers and oral mucositis, fibrotic diseases such as oral submucous fibrosis and radiation-induced fibrosis. To the best of our knowledge, silymarin-based oral mucoadhesive gel is not available for therapeutic use. However, a study done using silymarin cream for skin application is available in the literature, where its application in radiodermatitis of breast cancer patients, as a double-blinded placebo-controlled clinical trial, showed a significant delay in radiodermatitis development and progression in the silymarin group, with absence of notable side effects on the skin [8]. A study conducted using topical skin formulations of silymarin in melasma showed good results with minimal side effects on prolonged application for three months [13]. Silymarin also exhibited a gastroprotective effect in a study done by Sharma et al., in 2018, where silymarin-loaded beads of chitosan-MMT exhibited good mucoadhesion and efficient release of the drug, and were found to be a promising drug for the treatment of gastric ulcers [14]. Silymarin is known to be a safe drug with very minimal side effects. No major side effects or life-threatening complications have been reported so far in the literature, especially on prolonged and high-dosage use [15,16]. In 2011, Becher-Schiebe et al., conducted an observational study on 101 women with breast cancer who had had breast-conserving surgery, where they used an alternate, open, nonrandomized schedule to assign patients to the silymarin and standard of care (SOC) groups. This trial exhibited silymarin-based cream to be a promising candidate for a safe prophylactic treatment option of radiodermatitis [17].The absence of significant side effects, even upon long-term use makes it an ideal candidate for therapeutic use in oral inflammatory and fibrotic conditions, which warrants a longer duration of treatment and follow-up.

According to the literature survey, there is no silymarin-based mucoadhesive gel for oral topical application. The major hurdle is solubility, and the same is predicted to be overcome with formulation trials using a combination of newer polymers such as carbopol. Carbopol constitutes acrylic acid-poly alkenyl ether/divinyl glycol cross-linked primary polymer particles of about 0.2 to 6.0 micron average diameter with high molecular weight. Demonstrated to create a tenacious bond with the mucus membrane resulting in strong bio-adhesion, several commercial oral and topical products available today and under investigation have been formulated with carbopol polymers. Along with excellent adhesion forces, they lower the concentration of active ingredients and provide patient compliance with increased bioavailability of certain drugs. In the present study, silymarin gel was prepared using carbopol, propylene glycol, methylparaben, propylparaben, triethanolamine and peppermint oil. The stability, spreadability and release studies, *in vitro* antioxidant activity and *ex vivo* diffusion study using a chick embryo Franz diffusion model showed good promising results. These results are in concurrence with the previous study of silymarin gel for skin preparation, using the same ingredients, which demonstrated to have good viscosity, spreadability and antifungal properties with pH within the range of skin pH. The skin formulation also showed stability for up to two months at 40 °C, and had no skin irritation in human volunteers [7]. Among the three gel formulations prepared using pomegranate flowers by Aslan and his colleagues, one was preferred as a superior formulation because of its proper appearance and uniformity, acceptable viscosity, mucoadhesiveness and stability at different temperatures [9]. The present study of silymarin gel formulation trials resulted in an optimal gel formulation comparable to the gel obtained from the above-mentioned study with acceptable physicochemical parameters as shown in the results. Post six months of storage, no physiological alterations or changes of pH were observed. Non-fickian drug release and 22% drug permeation established its oral mucosal membrane permeability. Some of the physical methods to improve drug permeation include nanoformulations, liposomes, ethosomes, microemulsion, hydrogels, etc, which can be taken up in future studies [18].

The pH adjustment in the F10 was optimized maximally to 6.4, with further adjustment bearing on its viscosity. The normal salivary pH is 6.2–7.6, varying to 6–7 in various oral pathologies. A study performed by Foglio-Bonda et al., in 2017, reported that the salivary pH of 88 patients with oral lesions was 6.7 and the mean pH of 80 patients without oral mucosal lesions was 6.95 [19]. Oral disease severity, due to smoking and poor oral hygiene habits, is also strongly correlated with low salivary pH values (6.25 in stage IV periodontal disease) [20]. Hence, the pH of the oral mucosa tends to vary with oral disease presentations and habits such as tobacco.

A study employing ultrasonication (for better drug permeation) for the preparation of a silymarin-loaded nanostructured lipid carrier (NLC) gel showed antiproliferative, antioxidant, anti-inflammatory and antitumor activity in ex vivo and in vivo studies of skin cancer models [21]. The establishment of clinical efficacy of the gel shall be the impetus for further expansion of its therapeutic index, possibly as nano gel. Plans of preparation of a nano-based silymarin gel could probably increase the release percentage and offer better drug availability for improved efficacy. This formulation has set the pace for the development of the needed therapeutic option for oral diseases such as oral ulcers (aphthous ulcers and traumatic ulcers), oral mucositis and potentially malignant disorders such as oral submucous fibrosis, in the form of optimally bioavailable silymarin, thus potentializing its antifibrotic, anti-oxidant, anti-inflammatory and cytoprotective properties. Hence, multi-centric clinical trials on the topical application of silymarin mucoadhesive gel for different oral diseases can pave the way for identifying its therapeutic efficacy in different oral diseases.

## 4. Conclusions

Silymarin mucoadhesive gel was prepared using a carbopol base and was found to show optimum results with pre-formulation studies. The prepared silymarin oral mucoadhesive gels, formulation F10, had improved mucoadhesive properties, drug delivery and bioavailability to the oral mucosa, according to the physicochemical evaluation results. Our *ex vivo* analysis confirms formulation permeation and investigated the potential for oral mucosal delivery. The next step in the research process is to develop a nano gel using poly(lactic-co-glycolic acid) nanoparticles and conduct clinical trials in different oral pathologies.

## 5. Materials and Methods

The Serum Institute of India Pvt. Ltd. Pune, India supplied silymarin. Carbopol 934, methyl paraben and propyl paraben were purchased from Loba Chemie Pvt. Ltd. Mumbai, India. Propylene glycol and triethanolamine were obtained from SRL Chemicals Pvt. Ltd. Chennai, India.

### 5.1. Preparation of Silymarin-Based Mucoadhesive Gel

Silymarin, which is the bioflavanoid present in *Silybum marianum*, has poor water solubility. Because of its low bioavailability, mucoadhesive gels with optimized polymer combinations were planned as an improved silymarin formulation for potential use in oral mucosa as a topical preparation.

The gel was prepared by dispersing carbopol into distilled water to which propylene glycol had previously been added. Methylparaben and propylparaben were added to this precisely weighed quantity. Then, silymarin, which was previously solubilized in propylene glycol, was slowly added with gentle stirring in the carbopol gel base. Triethanolamine was added to neutralize the mixture and peppermint oil was added as a flavoring agent.

### 5.2. Pre-Formulation Studies

The color, odor, taste, and melting point of silymarin were determined. According to the certificate of analysis on the label, the purity was found to be 70% total silymarin. The standard stock solution was prepared in distilled water, and 100 mg of silymarin was accurately weighed and transferred to a 100 mL standard volumetric flask, which was then filled with distilled water to a volume of 100 mL. A sufficient aliquot of the standard stock solution (10 mL) was transferred to a 100 mL standard volumetric flask; the volume was then made up to 100 mL with distilled water. 1 mL, 2 mL, 4 mL and 5 mL was pipetted out, and the final volume was made up to 100 mL with distilled water to achieve concentrations of 1–5 µg/mL solutions. The absorbance was measured against distilled water at 287 nm (λ max silymarin) and the standard curve of concentration versus absorbance was plotted. The Fourier-transform infrared spectroscopy (FT-IR) spectra matching system was used to identify any potential chemical reactions between the drugs and polymers. It was scanned between 4000 and 600 cm^−1^.

### 5.3. Evaluation Parameters

#### 5.3.1. Viscosity

The measurement of the viscosity of the formulated gel was determined by a Brookfield Viscometer.

#### 5.3.2. pH

The pH of the gel formulation was determined by using a digital pH meter [22].

#### 5.3.3. Homogeneity

Visual inspection was used to verify the homogeneity of the formed gel formulation. They were examined for the existence of aggregates and the appearance of aggregates [23].

#### 5.3.4. Spreadability

The therapeutic effectiveness of the formulations was also determined by its spreadability. It was expressed in terms of the time it takes two slides to slip off the gel that is put between the slides, under the direction of a certain load. It was calculated using the following formula: S = M*L/t, where M denotes the weight attached to the upper slide, L denotes the length of the glass slide, and T denotes the time required to separate the slides [24,25].

#### 5.3.5. Drug Content

A specified amount of formulated gel was dissolved in 100 mL of pH 6.8 phosphate buffer. This solution was filtered and spectrophotometrically estimated at 287.0 nm with phosphate buffer (pH 6.8) as a blank.

#### 5.3.6. Stability Study

As per the I.C.H. guidelines, the shelf-life of the formulation was determined and any incompatibility within the formulation was identified as changes in appearance and drug content of the stored mucoadhesive gel, which was observed after 1, 2, 3, 4, 5 and 6 months [26].

#### 5.3.7. Drug Release Study by Open-Ended Cylinder

A glass cylinder measuring 10 cm in height, 3.7 cm in diameter, and 3.1 cm in diameter with both ends open were used. A glycerol-soaked cellophane membrane was attached to one end of the cylinder. It kept 5 g of the gel under investigation. At the receptor compartment, a beaker containing 100 mL of 6.8 pH buffer solution was used. The sample was submerged to a depth sufficient to keep it below the medium’s surface in the receptor compartment. While at 37 °C, the medium in the compartment was agitated with a magnetic stirrer. Every 10 min, 5 mL of the sample was removed and measured at 287 nm for 3 h. Every time, the volume extracted was replaced by an equivalent amount of medium [27]. The investigations were carried out in triplicate, and the standard deviation (SD) was determined.

#### 5.3.8. Drug Release Study by Diffusion Cell Apparatus

Phosphate buffer was used as the dissolution medium (pH 6.8). The membrane (DURAPORE HVLP 45 μm) was positioned over the receptor compartment. The receptor compartment was packed with 12 mL of dissolution medium and held at 37 °C. The dissolution medium was stirred with a magnetic stirrer at 50 rpm. At various intervals, 2 mL aliquots were removed and replaced with an equal volume of receptor medium [28]. The Electrolab diffusion cell apparatus was used for this study. The trials were carried out in triplicate, and the standard deviation (SD) was estimated.

#### 5.3.9. Release Kinetics

Dissolution and release of drugs are important phenomena for solid dosage forms such as tablets, capsules, and semisolid dosage forms such as creams, ointments, and implants, which deliver the drugs over the intended period ranging from hours to weeks and years. It is also applicable to the design and optimization of all kinds of modified release dosage forms such as sustained, delayed, controlled release dosage forms and novel drug delivery systems. For the evaluation of *in vitro* release kinetics to investigate the mechanism of release, the data were analyzed with the following mathematical models [29].

To investigate the mechanism of release, the data were analyzed with the following mathematical models: zero-order kinetic (Equation (1)), first-order kinetic (Equation (2)) and Higuchi kinetic (Equation (3)) Korsmeyer-Peppas model (Equation (4)), and Hixson-Crowell cube root law (Equation (5)) [30,31].

Q_t_ = K_0_t (1)

ln Q_t_ = ln Q_0_ − K_1_t (2)

Q_t_ = K_h_t^1/2^ (3)

Mt/Mα = K_p_t^n^ (4)

Q_0_ ^1/3^ − Q_t_ ^1/3^ = K_HC_t (5)

### 5.4. In Vitro Antioxidant Studies for Prepared Gel

#### 5.4.1. 2,2-Diphenylpicrylhydrazyl (DPPH) Free Radical Scavenging Test

Various concentrations of standard ascorbic acid and samples, namely 100, 200, 400, 800 and 1000 μg/mL, were prepared in distilled water. An equal volume of different concentrations of standards and DPPH were mixed separately in a clean and labelled tube and the tubes were incubated at room temperature in the dark for 30 min. The absorbance was measured at 517 nm using a UV-VIS spectrophotometer [32]. The percentage inhibition was calculated using the formula:

Percentage inhibition = (Abs control − Abs sample)/Abs control × 100

#### 5.4.2. Nitric Oxide Scavenging Assay

A 3 mL reaction mixture containing sodium nitroprusside (10 mM in phosphate-buffered saline) and various concentrations of standard ascorbic acid and samples, namely 100, 200, 400, 800 and 1000 μg/mL, was prepared in distilled water and kept in incubated at 37 °C for 4 h. To the incubation solution, 0.5 mL of Griess reagent was added and the absorbance was read at 546 nm [33]. The percentage inhibition was calculated using the formula:

Percentage inhibition = (Abs control − Abs sample)/Abs control × 100

### 5.5. Ex Vivo Permeation Study

An earlier study performed by El-Samaligy et al., in 2006, proposed that silymarin liposomal buccal delivery showed steady-state permeation through a chicken buccal mucosa for 6 hrs, and was higher than free drug powder. Hence, the drug permeation study was carried out through chicken buccal mucosa using a Franz diffusion cell. The chicken buccal mucosa was surgically removed from the underlying muscular layer by cutting the loose connective fibers, then cleaned from surface fats and washed with isotonic phosphate buffer at pH 6.8. The buccal mucosa, with the mucosal surface facing up, was placed between the donor and receptor compartments. The receptor compartment was filled with phosphate buffer (pH 6.8) and the diffusion cells were placed on hot plate-multiple magnetic stirrers at 37 °C and 150 rpm for 3 h. Samples were withdrawn at different intervals, filtered and replaced by fresh PB, and analyzed spectrophotometrically at wavelength 287 nm. The investigations were carried out in triplicate, and the standard deviation (SD) was determined [18,34].

## Figures and Tables

**Table 1:** Various parameters evaluated in the chosen silymarin gel formulation (F10).

S.No	Parameters	Observation
1	**Preformulation studies**	
	**Organoleptic characters**	Color: Yellow
		Odor: Characteristic
		Taste: Bitter
	Melting point	150 °C
	Linear regression analysis	It obeys Beers-Lamberts law
	FTIR spectroscopy	No interaction was observed
2	**Evaluation parameter for gel**	
	Organoleptic character	Color: Pale yellow color
		Odor: Mint odor
		Taste: Astringent taste
	pH	6.42
	Viscosity	3700 ± 0.98 to 7400 ± 0.32 cps
	Homogeneity	No visible particles are seen
	Spreadability	23.75
	Theoretical Drug content	32.77 ± 0.20 mg/g
	Stability	No change in physical properties was observed
	Drug release	Percentage cumulative drug release was found to be 2.6% and 3.25% for open-ended cylinder and diffusion cell, respectively, after 3 h
	Release kinetics	Zero order, first order, Higuchi kinetic
	*In vitro* antioxidant	Presence of antioxidant activity is confirmed
	*Ex vivo* diffusion study	In 3 h, it was found to be 3.03% and coefficient range was 0.9701

**Table 2:** Physicochemical evaluation for various gel formulations.

Evaluation Parameters		F1	F2	F3	F4	F5	F6	F7	F8	F9	F10
**pH**		4.2 ± 0.02	4.9 ± 0.04	5.0 ± 0.01	5.5 ± 0.03	5.7 ± 0.05	5.8 ± 0.04	6.0 ± 0.01	6.2 ± 0.04	6.3 ± 0.03	6.4 ± 0.01
**Homogeneity**		good	Good	good	good	good	good	good	good	good	good
**Spreadability**		10.73 ± 0.01	15.98 ± 0.03	18.01 ± 0.02	18.95 ± 0.04	19.73 ± 0.09	19.89 ± 0.01	20.56 ± 0.03	21.64 ± 0.02	22.05 ± 0.04	23.75 ± 0.03
**Viscosity (cpm)**		3700 ± 0.98	3490 ± 0.07	5000 ± 0.56	5231 ± 0.02	5409 ± 0.34	6000 ± 0.51	6302 ± 0.33	6589 ± 0.54	7000 ± 0.94	7400 ± 0.32
**Theoretical drug content (mg /g)**		0.2 ± 0.01 (Silymarin:0.5 g)	0.5 ± 0.21 (Silymarin:0.5 g)	0.87 ± 0.78 (Silymarin:0.5 g)	0.95 ± 0.12 (Silymarin:0.5 g)	1.9 ± 0.10 (Silymarin:0.5 g)	10.72 ± 0.76 (Silymarin:1 g)	12.1 ± 0.80 (Silymarin:1 g)	17.20 ± 0.37 (Silymarin:1 g)	29.83 ± 0.25 (Silymarin:1 g)	32.77 ± 0.20 (Silymarin:1 g)
**Stability** **(6 months at room temperature)**	**pH**	6.5 ± 0.03	6.5 ± 0.12	6.5 ± 0.15	6.6 ± 0.04	6.8 ± 0.21	6.8 ± 0.40	6.8 ± 0.31	6.9 ± 0.01	7.0 ± 0.04	7.0 ± 0.06
	**Drug content (mg /g)**	0.17 ± 0.19	0.23 ± 0.01	0.30 ± 0.83	0.52 ± 0.06	0.55 ± 0.15	1.9 ± 0.34	5.34 ± 0.57	9.8 ± 0.93	15.34 ± 0.05	28.00 ± 0.004

**Table 3:** Release kinetics for optimized formulation F10.

	Zero Order	First Order	Higuchi Diffusion	Korsmeyer Peppas
R^2^	0.9475	0.9475	0.9869	0.9448

**Table 4:** Ex vivo diffusion study of silymarin.

S.NO	Time (mins)	% Cumulative Drug Release
1.	10	0.057 ± 0.0
2.	20	1.52 ± 0.03
3.	30	2.66 ± 0.05
4.	40	4.11 ± 0.09
5.	50	6.05 ± 0.02
6.	60	8.32 ± 0.06
7.	90	11.05 ± 0.03
8.	120	14.03 ± 0.09
9.	150	17.56 ± 0.04
10.	180	21.97 ±0.18

## Data Availability

The data associated with the manuscript are available from the first and corresponding authors.

## Supplementary Materials

The following supporting information can be downloaded at: https://www.mdpi.com/article/10.3390/gels9020139/s1, Table S1: Composition of formulated silymarin mucoadhesive gel.
