# Supplementary material for: Preparation and Characterization of Silymarin Gel: A Novel Topical Mucoadhesive Formulation for Potential Applicability in Oral Pathologies
Source: Gels. 2023 Feb 7;9(2):139. doi: 10.3390/gels9020139 (PMC9956077; doi:10.3390/gels9020139)
Supplement: Supplementary file 1 [file gels-09-00139-s001.zip › gels-2166265-supplementary.pdf]

gels

Supplementary Materials

Preparation and Characterization of Silymarin Gel: A Novel Topical Mucoadhesive Formulation for Potential Applicability in Oral Pathologies

Divyambika Catakapatri Venugopal [1,†], Reshma Devi Senthilnathan [2,†], Yasasve Madhavan [1], Saba Maanvizhi [2], Sathasivasubramanian Sankarapandian [1], Vijayalakshmi Ramshankar [3,*] and Mangathayaru Kalachaveedu [4,*]

Table S1. Composition of formulated silymarin mucoadhesive gel.

Ingredients	F1	F2	F3	F4	F5	F6	F7	F8	F9	F10
**Silymarin**	**0.5g**	**0.5g**	**0.5g**	**0.5g**	**0.5g**	**1g**	**1g**	**1g**	**1g**	**1g**
**Carbopol 934**	0.1g	0.5g	1g	1.5g	2g	0.1g	0.5g	1g	1.5g	**2g**
**Propylene glycol**	50ml	50 ml	50 ml	50 ml	50 ml	50 ml	50 ml	50 ml	50 ml	**50 ml**
**Methyl Paraben**	0.001 g	0.001 g	0.001 g	0.001 g	0.001 g	0.001 g	0.001 g	0.001 g	0.001 g	**0.001 g**
**Propyl paraben**	0.01g	0.01g	0.01g	0.01g	0.01g	0.01g	0.01g	0.01g	0.01g	**0.01g**
**Triethanolamine**	0.5ml	0.5ml	0.5ml	0.5ml	0.5ml	0.5ml	0.5ml	0.5ml	0.5ml	**0.5ml**
**Distilled water**	50ml	50ml	50ml	50ml	50ml	50ml	50ml	50ml	50ml	**50ml**
**Pepper mint oil**	0.1ml	0.1ml	0.1ml	0.1ml	0.1ml	0.1ml	0.1ml	0.1ml	0.1ml	**0.1ml**
